# Using GPS-enabled cell phones to track the travel patterns of adolescents

**DOI:** 10.1186/1476-072X-7-22

**Published:** 2008-05-21

**Authors:** Sarah E Wiehe, Aaron E Carroll, Gilbert C Liu, Kelly L Haberkorn, Shawn C Hoch, Jeffery S Wilson, J Dennis Fortenberry

**Affiliations:** 1 Children's Health Services Research, Department of Pediatrics, Indiana University School of Medicine, 410 West 10th Street, HS1020, Indianapolis, IN, 46202, USA; 2 Regenstrief Institute, Inc, Indianapolis, IN, 46202, USA; 3 Department of Geography, Indiana University Purdue University Indianapolis, Indianapolis, IN, 46202, USA; 4 Section of Adolescent Medicine, Department of Pediatrics, Indiana University School of Medicine, Indianapolis, IN, 46202, USA

## Abstract

**Background:**

Few tools exist to directly measure the microsocial and physical environments of adolescents in circumstances where participatory observation is not practical or ethical. Yet measuring these environments is important as they are significantly associated with adolescent health-risk. For example, health-related behaviors such as cigarette smoking often occur in specific places where smoking may be relatively surreptitious.

**Results:**

We assessed the feasibility of using GPS-enabled cell phones to track adolescent travel patterns and gather daily diary data. We enrolled 15 adolescent women from a clinic-based setting and asked them to carry the phones for 1 week. We found that these phones can accurately and reliably track participant locations, as well as record diary information on adolescent behaviors. Participants had variable paths extending beyond their immediate neighborhoods, and denied that GPS-tracking influenced their activity.

**Conclusion:**

GPS-enabled cell phones offer a feasible and, in many ways, ideal modality of monitoring the location and travel patterns of adolescents. In addition, cell phones allow space- and time-specific interaction, probing, and intervention which significantly extends both research and health promotion beyond a clinical setting. Future studies can employ GPS-enabled cell phones to better understand adolescent environments, how they are associated with health-risk behaviors, and perhaps intervene to change health behavior.

## Background

Risky health behaviors contribute significantly to adult mortality [1]. Many of these behaviors are developed in adolescence and are preventable [2-4]. Numerous studies have shown that risk behaviors are associated with neighborhood context [5-12], yet these studies rely solely on residential address to define context. This may mis-specify contextual exposure as adolescents often spend more than half their time away from home [13]. Few studies report where adolescents spend time outside of their homes and school [13]. In addition, a census tract, block group, or other arbitrary geographic area surrounding a residential address may not accurately characterize where an individual lives, travels, and interacts with others [14]. In order to develop policies and interventions to promote a healthful environment, we must understand where adolescents spend time.

With the location tracking capabilities of global positioning system (GPS) devices, we may gather more detailed information on adolescents' travel. In addition, GPS-enabled devices such as cell phones allow a mode of contact with the study participant in several ways – monitoring study compliance, troubleshooting problems with the device or data collection, and collecting additional information pertaining to health-risk behaviors. This is a unique application of GPS-enabled cell phones and addresses several key limitations in research relating to contextual determinants of health. In a population where it is often difficult to assess accurate and reliable information on behaviors, GPS offers a timely, objective, and potentially more acceptable method of evaluating contextual exposure.

GPS technology has been used in a variety of applications to measure exposures or activities. GPS technology has greatly expanded the scope of space-time analyses by allowing the recording of not only trip origins and destinations, but also the routes traveled. Traditionally, GPS technology has been used for business and leisure applications: e.g. guiding agricultural machinery for planting and pesticide application [15], coaching for high performance athletes [16], and deploying hunters for the U.S. Forest Service [17]. More recently, GPS technology has shown promise in studying human behavior: e.g. the Lexington Area Travel Study [18] and Oklahoma Urban Air Toxics study [19]. The Lexington Area Travel Study conducted in the late 1990s collected information for more than 200 drivers whose movements were recorded over a 6-day period. It demonstrated the reliability of GPS technology and the feasibility for visualization and modeling in 3-D GIS. This led to the discovery of significant differences in travel patterns by gender and economic status of the participants [20]. Data on individual space-time activity patterns has been used to study and model differences in accessibility to a range of facilities, such as parks and stores, using realistic street network information in the GIS [21]. With improved technology resulting in smaller GPS devices, researchers are exploring diverse public health applications including studying physical activity in children[22] and pesticide exposure in migrant farm workers [23].

The application of this approach to tracking the space-time activity patterns of adolescents is a logical extension to adolescent health behavior research. In addition to a novel use of GPS technology, this approach overcomes conceptual and logistical challenges faced by previous studies of context and health, including relying on static representations of context by a residential address or on inaccurate self-reporting, prohibitively expensive data collection strategies, and inability to measure level, volume, and pattern of activity [24]. We often do not know the specific places where health-related behaviors such as healthy diet, cigarette smoking, drug use, and unprotected sexual intercourse occur. Collecting data on these places may be difficult as they are relatively surreptitious and not conducive to detailed self-report. As such, devices such as GPS-enabled cell phones may aid in locating where these behaviors occur and better define the paths and events preceding them. Little is known, however, about whether it is feasible to use these devices for this purpose among adolescents. For example, we do not know whether adolescents will carry the phones with them if they know they are being tracked, what the data accuracy and reliability are given adolescent phone usage, charging habits, and overall study compliance; and if the device could also be used to collect information using a text-message-based survey instrument.

Thus, our overall objective was to test the feasibility of using GPS-enabled cell phones to track an adolescent's travel patterns and gather daily diary data. Specific objectives included (a) investigating how accurately and reliably the device collected and transmitted GPS coordinates, (b) determining whether the device could be used to gather survey data remotely on a daily basis, (c) assessing whether adolescents perceived being tracked for research purposes as acceptable, and (d) gaining preliminary insight into travel patterns among our study population.

## Methods

### Study population

The study enrolled 15 adolescent women from a clinic-based setting with the following inclusion criteria: (1) female, (2) age 14 to 17 years, (3) willing and able to travel at least 3 blocks away from home at least once each day, (4) capable of carrying a phone with her at all times for a period of 7 days, and (5) understands and speaks English. We did not collect sociodemographic data from the young women that we recruited. According to the electronic medical records for the clinic from which we recruited, there were 1004 outpatient visits in which patients were young women 14 to 17 years old. The patients' race/ethnicity reported in these records was 32% white, 27% black, and 40% Latina. The majority (88%) were on public insurance. We obtained assent from each participant and consent from her parent/guardian. We staggered enrollment such that one to four women had phones in the field at a time so that we could modify the study protocol and cell phone programs in response to problems that arose. The research received approval from the Institutional Review Board of Indiana University – Purdue University at Indianapolis.

### Procedures and measures

#### Orientation meeting procedure

A research assistant (RA) met the adolescent at a location of her choosing. The RA oriented the adolescent to the phone – how to turn it on, charge it, and make calls. The RA answered any questions that the adolescent had and reviewed the study requests:

1. Carry the phone with you at all times while awake

2. Always keep the phone 'on'

3. Charge the phone overnight each night

4. Travel at least 3 blocks away from home at least once each day

5. Contact the RA in the event of malfunctioning or lost study equipment (the phone service agreement includes an insurance policy providing for free replacement in the event of loss or damage)

The adolescent could make unlimited calls in the evenings and weekends. She could also use up to 150 minutes of calls between 7 am and 7 pm during the weekdays of the study period.

#### Exit interview/process measures

At the conclusion of the 7-day participation period, the RA collected the phone and charging devices and conducted a semi-structured interview. Next, the RA reviewed the maps created from the GPS data and asked the adolescent to comment on whether she thought the map accurately represented her locations.

Process measures included:

1. Self-reported compliance in carrying the phone

2. Self-reported compliance in charging the phone

3. Self-reported accuracy of the maps in reflecting her 7 day location data

4. Periods in which the phone had no movement for over 24 hours

5. Number of phone malfunctions

6. Whether phone was used

7. Whether the phone was returned in working order

#### GPS-enabled cell phone

Participants carried Blackberry 7520 GPS-enabled cell phones (cost $150/phone). The cell phone measures 11.4 (L) × 7.4 (W) × 2.8 (D) centimeters, weighs 174 grams. The phones use an assisted-GPS system in which an approximate location is identified using less accurate cell phone towers and does not need a clear view of the sky to determine location. These cell phone tower data are subsequently processed to identify a more accurate point using satellite communication. This method is desirable because it uses less battery power (allowing a phone to remain charged for at least a 16 hour period of heavy use). Accuracy of positional determination is variable depending on city and location within a city but is reported to be approximately 6 meters horizontally and 10 meters vertically.

#### GPS data collection

Using the BlackBerry Java Development Environment, a background module was created that ran on startup and in the background. It was therefore invisible to the phone user and could not easily be turned off. At 5 minute intervals, the program obtained a "fix" on the participant's location and, through an http call, transmitted the location coordinates, battery level, device ID, and timestamp to a java servlet running on a server dedicated to the study. All data were stored in a MySQL database. Another application monitored the database and sent an SMS message to the phone if no diary data had been sent in the last day. A similar message was sent to the researchers if no diary data had been sent in two days. The program also provided the research assistant with a summary of GPS data for each phone so that she could contact adolescents from whom data was not being regularly sent.

#### Diary data collection

We instructed the participant to answer several questions each evening before going to bed using a program developed for the Blackberry platform. We were not interested in the answers to the questions but rather whether participants could answer various question types easily using a Blackberry and would reliably answer them on a daily basis. As such, we have devised questions which would elicit different modes of answering (yes/no, multiple choice pull-down menu, free text response). We also asked for comments regarding the phone or the study as an additional process measure. We asked the following questions:

What time did you wake up this morning? (ENTER TIME USING CLOCK OR CALENDAR)

Did you eat lunch today? (YES/NO)

YES → Approximately what time did you eat?

Did you charge the phone last night? (YES/NO)

NO → Did the phone's battery die today?

Did you need to charge the phone during the day today?

Where were you at 4 pm today? (HOME/SCHOOL/FRIEND'S HOUSE/SHOPPING/OUTSIDE IN THE NEIGHBORHOOD/OUTSIDE BUT NOT IN THE NEIGHBORHOOD/OTHER – PLEASE SPECIFY)

What color shirt are you wearing? (KEY ENTER)

Comments or notes on the study or phone: (KEY ENTER)

[The indented questions were skip pattern questions.] These data were transmitted to a secure server in a similar manner as the GPS data when the participant pressed 'send' at the end of the diary entry.

#### Reimbursement for participation

Adolescents were reimbursed $20 cash following agreeing to participate and $100 cash after completion of the exit interview.

### Data interpolation

We anticipated times when cell phones might encounter GPS signal reception interference (e.g. near power substation transformers) or obstruction (being enclosed in thick-walled building). When possible, we interpolated position in the event of lost GPS reception. Interpolation is a method in which missing spatial data are imputed using adjacent valid values. If there were significant temporal discrepancies between serial "fixes" on location, we recorded a "no data" observation.

When more than 5 minutes but less than 1 hour elapsed between measured data points, we imputed interim 5-minute time points. If 2 temporally adjacent points bounding a period of missing data were within 30-meters (the distance used to determine the 2 points were in the same or similar location), we assigned the missing GPS data point to the earlier point. For data points more than 30 meters apart, we imputed in 2 ways. In one case, we used the point which was closer to home and, in another, used the point which was further from home. Since results were very similar for these 2 approaches, we present the data using the closer imputed point. We performed all analyses by varying the time lapse cut-off for imputation (1 hour to 24 hours) and found similar results.

## Results

### Reliability and accuracy of the GPS-enabled cell phone to obtain location data

Most issues influencing the reliability of the GPS-enabled cell phones to obtain location data were related to user error. Among the 15 participants, there was a potential total of 100 days of data collection by carrying the GPS-enabled phone. Two adolescents forgot to carry the phone for 1 day each, a third left it at home for 1 day to recharge the battery, and the fourth was unable to bring it with her to school (5 days). There were 2 nights when the adolescent neglected to charge the phone, and the battery subsequently ran out the following day.

In addition to user error, there were several technical issues with the phones relating to the GPS functionality, particularly at the beginning of the study. There was only 1 adolescent whose GPS feature tracked her whereabouts for the entire 7 days without fail. For the rest of the adolescents, however, the failures were recognized remotely, and data collection failure was remedied within 1 day.

Of the GPS point locations collected, 83% were within 5–6 minutes of the previous point. Approximately 2% of points had more than a 1-hour lapse, and 0.5% more than a 4-hour lapse. In total, 71% of the possible 5-minute time points were occupied with GPS data transmitted directly from the cell phone, and 11% of the remaining missing data were between 2 known points 30-meters apart or less. Very often, the phone would stop transmitting a signal from within a building, including where the adolescent lived or went to school (according to participants' report when reviewing maps).

The 14 participants who completed an exit interview confirmed the accuracy of the maps created from their GPS data points. Two participants exhibited surprise when they saw how accurately the maps reflected where they traveled. Anecdotal accounts revealed the GPS device accurately portrayed the street they traveled on but not necessarily with complete accuracy. While the participants easily recognized the general area they were shown, and confirmed they were indeed there, there often were occasions that the points were not completely reflective of their exact location. This was especially true in neighborhoods, where homes are very close together and the points sometimes placed the girls in neighboring houses, rather than where they actually were.

None of the participants had difficulty reading the aerial photography or street maps, although most preferred the photography. Most first recognized a location on the map at 1:400 to 1:4000 scale and, after being oriented, had no difficulties identifying locations where GPS data were collected.

### Feasibility of gathering daily diary data remotely

For the first 8 young women who participated in the study, frequent contact had to be made with reminders to submit the diary entries daily. The reason for this seeming non-compliance was discovered to be a software issue. We corrected the software program and gave additional instructions to the subjects during orientation. Timely diary entries were received daily from the remainder of the adolescents. There were no difficulties with any of the question types (yes/no, multiple choice pull-down menu, or free text response).

While not all felt comfortable enough with the technology to regularly use the internet and/or texting functions, each young woman was able to make diary entries, as well as reply to text messages sent to them by the RA.

Because a maximum of four subjects were carrying phones at any one time, the RA spent 20–25 hours per week on average coordinating this study. Communication with subjects, once they were enrolled and carrying a phone was done almost exclusively via text messaging, which allowed the research coordinator to respond at her convenience. The amount of text messages received from the subjects ranged from one for the whole week to 4–5 per day. The only aspect of the study design that afforded any extra inconvenience was the timing of the appointments. With most of the girls in school during the day, both orientation meetings and exit interviews had to be held during the evening. This required the RA to work outside of normal business hours.

### Acceptability of being tracked for research purposes using GPS-enabled cell phones

Every adolescent/guardian pair invited to participate agreed to enroll in the study. All 15 participants completed the GPS-monitoring period. Contacting subjects during their study period was easy and successful. All phones were returned in a timely manner, per study protocol, and all were in complete working order and excellent condition when received by the RA.

Nearly all of the young women interviewed reported that the GPS feature of the phone was neither concerning nor did it influence their activity patterns or behavior. Three discussed the additional safety they felt knowing that their location could easily be determined if they were in danger. Many parents felt the same.

In contrast, several of the adolescents' peers felt threatened by the GPS tracking on the phone. During their exit interviews, 3 adolescents offered discussion about how their friends would not allow them to make calls from the phone because they thought they would be recorded or their activities would be reported to the police.

Many adolescents took advantage of the features of the phone to their own benefit. One young woman used the internet capability to download help with school work, while another used it to call for help when her car broke down, and she became stranded. A third adolescent explained how she and her friends became lost in a rural county and used her phone's internet capabilities to retrieve correct directions. Each adolescent used her phone's calling capabilities at least once per day.

Field notes collected by the RA during the initial interviews with the participants described how several of the adolescents were anxious to have the "free phone" and be given nearly unlimited calling capabilities for a week. Many of the young women took full advantage of this incentive by giving their phone number to peers and altering their voice mail messages to reflect their own names and personalities. This benefit to the participants was further confirmed during the exit interviews, when the young women who agreed to participate in future versions of GPS-tracking studies stated that as an incentive item, the phone was sufficient.

### Variability and distance in travel patterns among adolescent women

**Figure 1:**
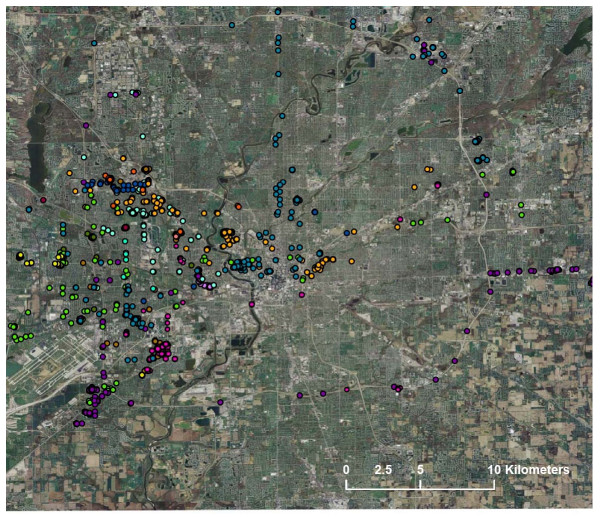
Aerial Photograph of Marion County with 15 Subjects' GPS Data.

The GPS data collected in this study show that travel patterns are variable in distance and direction throughout the metropolitan area, as are presented on an aerial photograph of Marion County, Indiana (Figure 1). Each subject has a different color dot depicting her GPS data points. This figure shows that study subjects' space-time paths vary widely across the city, and subjects spend time outside of what has traditionally been defined as "neighborhood". [Data on individual subjects at higher levels of magnification or designating home or school locations may be a breach of confidentiality and so has been deferred to preserve appropriate statistical disclosure control.]

**Figure 2:**
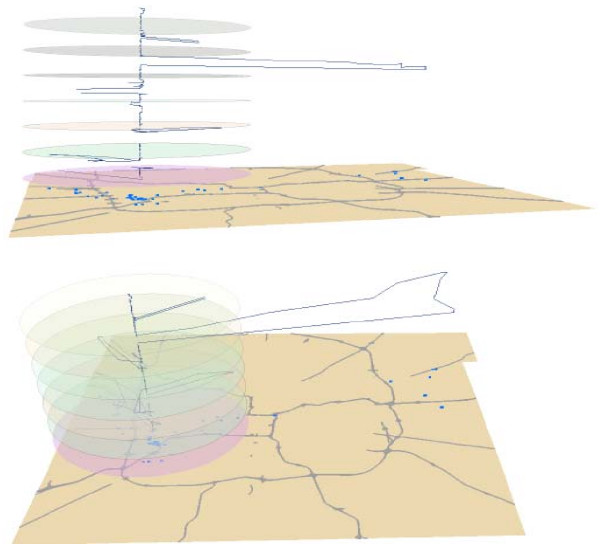
Example of space-time path in GIS.

There is also significant variability from day-to-day in distance from home and direction of travel. Figure 2 displays a 3-D representation of space-time paths from one pilot study participant. In the figure, the vertical axis represents time over the period of 7 days with division between days (12:00 am) denoted by horizontal discs. The boundaries of the horizontal plane represent the spatial scope of the analysis, in this case Marion County. The blue line represents the space-time path connecting GPS waypoints.

**Figure 3:**
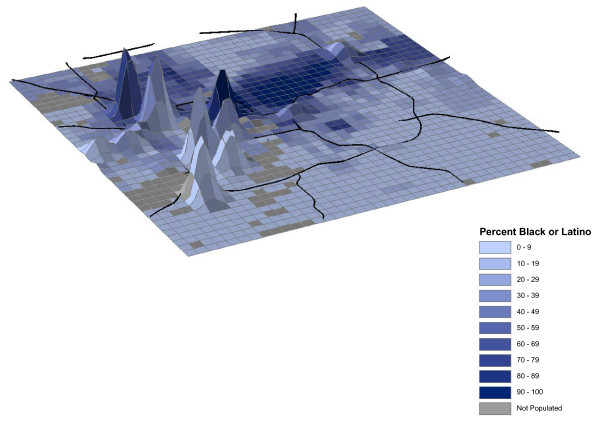
Time density map of Marion County by percent of black or Latino population.

Individual space-time activity patterns can be visualized using 3-D tools in order to map their summary as density plots. Time density maps depict time on the z-axis for all participants' data points in Marion County, Indiana. Using aggregated (and imputed using straight-line methods which equally distributes a Euclidian distance between 2 points depending on the number of time points with no data recorded, assigning a set of geographic coordinates along this Euclidian path) data from all participants, we show variation in location attributes where participants spend time (Figure 3). This map depicts the time (z-axis) spent in areas as described by minority concentration. Attribute data derived from 2000 U.S. Census and land use data are shown in grid cells 805 meters × 805 meters in dimension (approximate 0.25 square miles) as implemented in the Twin Cities Walking Study GIS Protocols [25]. Similar analyses can be done using other attribute data, including population density, household wealth, or concentrations of crime, liquor outlets, or health care establishments.

## Discussion

This preliminary study documents the feasibility of using GPS-enabled cell phones to track travel patterns of adolescent women. It confirms that cell phones can accurately and reliably track the locations of participants, and participants can use the cell phone as a diary device to record daily behaviors. The cell phone acted both as a potent incentive and promoted compliance in consistently being carried by the participants. Participants denied that the GPS-tracking influenced their activity patterns.

Analysis of the pilot data suggests that most of the participants have variable paths extending beyond their immediate neighborhoods. In cases in which participants stayed primarily within their neighborhood as well as when they traveled out of their neighborhood, it demonstrated the capability of collecting more detailed information of where they spent time.

Determinants of adolescent health-risk behaviors continue to elude researchers and clinicians. Our current research in health-risk behaviors is limited by the inability to directly observe these acts due to social constraints and privacy issues. We know that these behaviors are socially based, however, and tend to cluster geographically and among certain high-risk individuals [5,7,9,26-31]. Using GPS technology, we may gain unprecedented insight into where adolescents spend time. By linking their travel patterns with reports of their health-related behavior, we may better intervene to more effectively promote resilience and prevent adverse health outcomes among adolescents.

Technologies such as GPS-enabled cell phones are increasingly encountered by adolescents in their daily lives and used by commercial entities and by parents. In addition, our experience shows that, with due attention to privacy and confidentiality issues, this method is acceptable to teens and can be successfully employed without evidence of compromise to privacy or confidentiality. Like all new methods, anticipation of potential misuses of information, implementation of safeguards, and careful monitoring for unanticipated effects are critical. Thus, we have strictly followed standard operating procedures for research which collects sensitive and detailed data. We opted to disclose both the purpose and intentions of this research to the participant and her parent/guardian. Although the lack of deception may contribute to differential use of the technology (despite this being denied by the adolescent), we thought it unethical to propose any other approach. We stated up front that these data would not be shared with anyone outside of the research team, except in the case where the GPS data would offer protection to someone in imminent harm. This can be achieved more securely with a Certificate of Confidentiality from the National Institute of Health. We sought every opportunity to protect the data by implementing multiple data security measures. For example, the GPS data were not stored on the cell phone but sent directly to a secure server. The server and all computers which have access to the data are protected at multiple levels with secure passphrases, data encryption, and physical barriers such as containment within locked offices with limited access. Finally, we closely monitor these and future data with the help of an independent data review committee with whom we meet quarterly. In sum, we have implemented procedures to maximally protect the participant's privacy and fully promote the highest ethical standards for research with GPS data and sensitive health information.

This study offers several extensions to our current understanding of GPS tracking. First, to our knowledge, this is the first study to utilize GPS-enabled cell phones to collect location information for contextual health research. Previous studies have relied on GPS units that store data for subsequent downloading whereas cell phone GPS units can transmit data on an ongoing basis. Continuous transmission of data has several advantages including the ability to more frequently monitor data collection, intervene in cases of participant non-compliance or equipment error, and opportunity to retrieve partial data in the event of power loss or device loss, damage, or malfunction. A potential barrier with such devices is a reluctance to have one's location being continuously monitored; however, in interviews, none of the subjects reported this potential loss of privacy as a deterrent to research participation. Conversely, we observed that the cell phone served as an incentive for participation. Finally, cell phone devices can serve as a platform for supplemental data collection via either instant messaging or voice surveys.

A second contribution of this study is that it expands our knowledge regarding travel patterns for adolescent females. Adolescence represents a vulnerable time in which risk-taking behavior increases [32] which is highly correlated with increased morbidity in later adolescence and adulthood [1,33]. This age range also represents a time when children often become increasingly independent from caregivers, widening their travel patterns and contextual exposures. Of note, previous studies have focused on adult drivers or younger children and to our knowledge, this is the first study that focuses on adolescent travel patterns. Elgethun *et al*. have published several articles describing use of GPS to assess exposure of children ages 3 to 5 years old to pesticides [23]. Mackett *et al*. have also employed GPS to analyze factors relating to mode of transit used by children to travel to school [34]. Studies of adult travel patterns using GPS technologies have occurred across a wide range of disciplines including public health [35], geography [36,37], urban planning [38], and women's studies [20,39]. In each of these instances of research employing GPS, investigators have found similar results to this project in that GPS has served as an accurate and reliable modality for obtaining detailed location/travel data that offers many advantages over approaches that rely on self-report.

A third contribution of this study is the method described for interpolating location. Several studies have discussed the issue of missing location data due to factors such as obstructed GPS signal, but methods for addressing missing location data are not well-described. The data interpolation method that we used made several assumptions. First, we assumed that there were no systematic factors which influenced when the phone would transmit GPS data points. In other words, gaps in GPS data transmission were random – *not *determined by participant censoring (turning the phone or GPS program off during periods in which they did not want to be tracked) or cell phone battery life and charging patterns (if the battery was more likely to die at the end of the day, for example). Participants denied changing their behaviors as a result of being tracked. Likewise, they denied censoring our ability to track them by manipulating the phones' tracking abilities. Participants reported regularly charging the phones. We also monitored this through an additional program which transmitted the remaining battery life at regular intervals to our secure server. The gaps in recorded data could have been due to physical obstruction or placement of cell phone towers. Although this is perhaps not random, this systematic bias would likely not be correlated with participant health-related behaviors. Second, we assumed that the missing data represented locations and paths between two registered GPS points. We had no way of determining where travel occurred between the two known points, what the rate of travel was, whether there was more time spent in the earlier or later point, and whether there were stops along the way. As such, it would be difficult to use this as an accurate method to calculate physical activity. For example, if an individual's data revealed that she was at her home at 4:00 pm and at the mall at 8:00 pm, we did not know if she was at home until 7:45 pm and then drove to the mall, if she drove to the mall at 4:05 pm and arrived by 4:20 pm, whether she walked to the mall over the course of 4 hours, or whether she left home sometime after 4:00 pm, went out to dinner en route to the mall, and arrived at the mall sometime before 8:00 pm. We addressed the issue of missing GPS data through a sensitivity analysis of different data interpolation trials. As described in the methods, we imputed the missing data as the point closer to home in one trial and as the point further from home in another trial. We found little difference in the results. In addition, we considered several cut-points at which data were imputed or excluded from analysis. These cut-points ranged from 1 hour to 24 hours. Since the majority of missing data were in the less than 1 hour range, we found little difference in our results using various cut-points. We were unable to account for potential stops that were not geographically positioned along the Euclidian path between the two known locations. We would have found, however, greater differences in our sensitivity analysis of cut-off points if this were frequently the case.

Finally, it is important to note that we found no off-the-shelf products to gather and use GPS data from cell phones in near real time. However, our project showed the feasibility of using free software development environments and platforms to create a system to both send and receive GPS data from Blackberry phones. Although technical issues occurred throughout the study, they were all overcome, and by the study's conclusions the system was operating robustly and consistently.

## Conclusion

GPS-enabled cell phones offer a feasible and, in many ways, ideal modality of monitoring the location and travel patterns of adolescents. In addition, it allows space- and time-specific interaction, probing, and intervention which significantly extends both research and health promotion beyond a clinical setting. We will continue using these devices to conduct research monitoring spatial and temporal variation in contextual exposure. Next steps include monitoring larger numbers of adolescent women, repeatedly over a year, to investigate differences in travel patterns by whether they engage in various health-risk behaviors. We anticipate rich data that will contribute to our understanding in both conceptual and technological domains. In future studies, we hope to use this platform to build space- and time-specific interventions.

## Competing interests

The authors declare that they have no competing interests.

## Authors' contributions

SEW developed the concept and design of the study, oversaw the acquisition of the data, was actively involved in the analysis and interpretation of the data, and drafted the manuscript.

AEC developed the software and helped in writing the methods section and gave substantive feedback on the remainder of the manuscript. GCL was actively involved data analysis and in the revising the manuscript. KH was the study coordinator and managed every aspect of the protocol including recruiting and consenting subjects and their guardians, orienting them to the study, troubleshooting problems that arose during the data acquisition, performing all exit interviews, and helping to interpret the data and revise the manuscript. SCH helped to impute missing geospatial data, perform the GIS analyses, and revise the manuscript. JSW and JDF made substantial contributions to the concept and design of the study and gave insightful revisions of the manuscript. All authors read and approved the final manuscript.

## References

[B1] Mokdad AH, Marks JS, Stroup DF, Gerberding JL (2004). Actual causes of death in the United States, 2000. JAMA.

[B2] Chassin L, Presson CC, Sherman SJ, Edwards DA (1990). The natural history of cigarette smoking: predicting young-adult smoking outcomes from adolescent smoking patterns. Health Psychol.

[B3] Hawkins JD, Catalano RF, Kosterman R, Abbott R, Hill KG (1999). Preventing adolescent health-risk behaviors by strengthening protection during childhood. Arch Pediatr Adolesc Med.

[B4] Zapert K, Snow DL, Tebes JK (2002). Patterns of substance use in early through late adolescence. Am J Community Psychol.

[B5] Lee RE, Cubbin C (2002). Neighborhood context and youth cardiovascular health behaviors. Am J Public Health.

[B6] Cubbin C, Hadden WC, Winkleby MA (2001). Neighborhood context and cardiovascular disease risk factors: the contribution of material deprivation. Ethn Dis.

[B7] Duncan SC, Duncan TE, Strycker LA (2002). A multilevel analysis of neighborhood context and youth alcohol and drug problems. Prev Sci.

[B8] Ross CE (2000). Walking, exercising, and smoking: does neighborhood matter?. Soc Sci Med.

[B9] Diez Roux AV, Merkin SS, Hannan P, Jacobs DR, Kiefe CI (2003). Area characteristics, individual-level socioeconomic indicators, and smoking in young adults: the coronary artery disease risk development in young adults study. Am J Epidemiol.

[B10] Diez-Roux AV, Kiefe CI, Jacobs DR, Haan M, Jackson SA, Nieto FJ, Paton CC, Schulz R, Roux AV (2001). Area characteristics and individual-level socioeconomic position indicators in three population-based epidemiologic studies. Ann Epidemiol.

[B11] Karvonen S, Rimpela A (1996). Socio-regional context as a determinant of adolescents' health behaviour in Finland. Soc Sci Med.

[B12] Cubbin C, Santelli J, Brindis CD, Braveman P (2005). Neighborhood context and sexual behaviors among adolescents: findings from the national longitudinal study of adolescent health. Perspect Sex Reprod Health.

[B13] Larson RW, Richards MH, Sims B, Dworkin J (2001). How urban African American young adolescents spend their time: time budgets for locations, activities, and companionship. Am J Community Psychol.

[B14] Coulton CJ, Korbin J, Chan T, Su M (2001). Mapping residents' perceptions of neighborhood boundaries: a methodological note. Am J Community Psychol.

[B15] Holton W (2000). Farming from a new perspective: remote sensing comes down to earth. Environ Health Perspect.

[B16] Liebermann D, Katz L, Hughes M, Bartlett R, Mcclements J, Franks I (2002). Advances in the application of information technology to sport performance. Journal of Sports Sciences.

[B17] Lyon L, Burcham M (1998). Tracking Elk Hunters with the Global Positioning System. USFS RMRS-RP-3.

[B18] Battelle Transportation Division (1997). Global Positioning System for Personal Travel Surveys: Lexington Area Travel Data Collection Test, Final Report. Federal Highway Administration.

[B19] Phillips M, Hall T, Esmen N, Lynch R, Johnson D (2001). Use of global positioning system technology to track subject's location during environmental exposure sampling. J Expos Anal Environ Epidemiol.

[B20] Kwan M (1999). Gender and Individual Access to Urban Opportunities: A Study Using Space-Time Measures. The Professional Geographer.

[B21] Miller H (1999). Potential contributions of spatial analysis to geographic information systems for transportation (GIS-T). Geographical Analysis.

[B22] Children's Activities, Perceptions and Behaviour in the Local Environment (CAPABLE). http://www.casa.ucl.ac.uk/capableproject/.

[B23] Elgethun K, Fenske RA, Yost MG, Palcisko GJ (2003). Time-location analysis for exposure assessment studies of children using a novel global positioning system instrument. Environ Health Perspect.

[B24] Cohen-Hubal E, Sheldon L, Burke J, McCurdy T, Berry M, Rigas M, Zartarian V, Freeman N (2000). Children's exposure assessment: a review of factors influencing children's exposure, and the data available to characterize and assess that exposure. Environ Health Perspect.

[B25] Forsyth A (2006). Environment and Physical Activity: GIS Protocols. Work In Progress, Version 40.

[B26] Cohen D, Spear S, Scribner R, Kissinger P, Mason K, Wildgen J (2000). "Broken windows" and the risk of gonorrhea. Am J Public Health.

[B27] Diehr P, Koepsell T, Cheadle A, Psaty BM, Wagner E, Curry S (1993). Do communities differ in health behaviors?. J Clin Epidemiol.

[B28] Gorman DM, Speer PW, Gruenewald PJ, Labouvie EW (2001). Spatial dynamics of alcohol availability, neighborhood structure and violent crime. J Stud Alcohol.

[B29] Kawachi I, Berkman LF, eds (2003). Neighborhoods and health.

[B30] Leventhal T, Brooks-Gunn J (2000). The neighborhoods they live in: the effects of neighborhood residence on child and adolescent outcomes. Psychol Bull.

[B31] Nelson MC, Gordon-Larsen P, Song Y, Popkin BM (2006). Built and social environments associations with adolescent overweight and activity. Am J Prev Med.

[B32] Centers for Disease Control and Prevention (2006). Youth Risk Behavior Surveillance – United States, 2005. Surveillance Summaries, June 9, 2006. MMWR.

[B33] Ezzati M, Lopez AD, Rodgers A, Hoorn S Vander, Murray CJ (2002). Selected major risk factors and global and regional burden of disease. Lancet.

[B34] Mackett R, Gong Y, Kitazawa K, Paskins J (2007). Children's local travel behavior – how the environment influences, controls, and facilitates it. 11th World Conference on Transport Reseach.

[B35] Rodriguez DA, Brown AL, Troped PJ (2005). Portable global positioning units to complement accelerometry-based physical activity monitors. Med Sci Sports Exerc.

[B36] Kwan M (2004). GIS methods in time-geographic research: geocomputation and geovisualization of human activity patterns. Geogr Ann.

[B37] Kwan M, Lee J, Goodchild M, Janelle D (2004). Geovisualization of Human Activity Patterns Using 3D GIS: A Time-Geographic Approach. Spatially Integrated Social Science.

[B38] Lindsey G, Han Y, Wilson J, Yang J (2006). Neighborhood Correlates of Urban Trail Use. Journal of Physical Activity and Health.

[B39] Kwan M (2000). Gender differences in space-time constraints. Area.

